# Reply to Muzzioli et al.: Communicating nutrition and environmental information to food system stakeholders

**DOI:** 10.1073/pnas.2302165120

**Published:** 2023-04-17

**Authors:** Michael Clark, Marco Springmann, Mike Rayner, Peter Scarborough, Jason Hill, David Tilman, Jennie I. Macdiarmid, Jessica Fanzo, Lauren Bandy, Richard A. Harrington

**Affiliations:** ^a^Nuffield Department of Population Health, University of Oxford, Oxford OX3 7LF, UK; ^b^Oxford Martin School, University of Oxford, Oxford OX1 3BD, UK; ^c^Department of Zoology, Interdisciplinary Centre of Conservation Science, University of Oxford, Oxford OX1 3SZ, UK; ^d^Smith School of Enterprise and Environment, University of Oxford, Oxford OX1 3QY, UK; ^e^National Institutes for Health and Care Research (NIHR) Biomedical Research Centre at Oxford, University of Oxford, Oxford OX3 7LF, UK; ^f^Department of Bioproducts and Bioengineering, University of Minnesota, St Paul, MN 55108; ^g^Department of Ecology, Evolution, and Behavior, University of Minnesota, St Paul, MN 55108; ^h^Bren School of Environmental Science & Management, University of California, Santa Barbara, CA 93117; ^i^The Rowett Institute, University of Aberdeen, Aberdeen AB25 2ZD, UK; ^j^Nitze School of Advanced International Studies, Johns Hopkins University, Washington, D.C. 20036; ^k^Global Food Ethics and Policy Program, Johns Hopkins University, Baltimore, MD 21205; ^l^Nuffield Department of Primary Care, University of Oxford, Oxford OX2 6GG, UK

Understanding how to effectively communicate the nutrition and environmental impacts of food products to food system stakeholders, such as consumers, retailers, civil servants, and policy-makers, is integral to transitioning toward sustainable and healthy food systems.

In their letter, Muzzioli et al. (1) raise two main points: 1) the diffuse relationship between nutrition and environment in figure 4 of ref. 2, introduces the possibility for trade-offs between these outcomes and 2) the functional unit that should be used report nutrition and environmental impacts of food products. Both have been discussed in depth in ref. 2 and elsewhere (3).

On the first point: The diffuse relationship between nutrition and environment shown in figure 4 is the reality of the food environment in which many of us live. Whilst there is a general trend for more nutritious foods to be more sustainable across the thousands of food products many of us are fortunate to be able to choose between, there are many outliers to this trend (e.g., table condiments, desserts, etc). This builds on findings in previous analyses focusing on commodities (4) or diets (5, 6), which despite their small sample size (often <15 data points) also found evidence of outliers to general nutrition–environment trend. This included commodities such as sugar-sweetened beverages (sustainable but unhealthy), certain types of fish (unsustainable but healthy), and diets primarily composed of these foods.

On the second point: The unit used to report the nutrition and environmental impacts of food should reflect how food system stakeholders make their decisions. As discussed in ref. 3, outcomes were reported per 100 g for two reasons: first, because most Nutrient Profiling Models (NPMs), including the NPM used in the analysis (7), were designed to assess outcomes per 100 g; second, the 100 g unit is used to set nutrition policy in many countries and also to report the nutrition content of food products. This means that food system stakeholders are familiar with seeing nutrition reported per 100 g, which reduced potential communication barriers. However, the 100 g unit is not perfect: it is not indicative of the amount typically consumed in a meal, but neither are pack sizes or serving sizes. For example, the serving size of UK ready meals ranged from 18 to 850 g (3). Until NPMs can be applied on a per-serving basis and until serving sizes are more regulated and standardized, we believe reporting outcomes per 100 g will remain a more robust approach than using serving sizes.

As mentioned in ref. 1, future research should investigate how complex and often conflicting environmental and nutrition information can be jointly communicated to motivate healthier and more sustainable food decision-making. This includes the unit used to report food-related impacts, how these impacts are communicated (front-of-pack labels, certification schemes, etc.), and how to communicate them in a way that does not increase existing food disparities.
